# Circadian Rest/Activity Rhythms and MRI-Derived Measures of Brain Health in Older Adults

**DOI:** 10.1093/geroni/igaf122.1955

**Published:** 2025-12-31

**Authors:** Adam Spira, Lacey Etzkorn, Amal Wanigatunga, Wendy Wang, Bruce Wasserman, Daniel Callow, Jennifer Schrack, Lin Yee Chen

**Affiliations:** Johns Hopkins Bloomberg School of Public Health, Baltimore, Maryland, United States; Johns Hopkins Bloomberg School of Public Health, Baltimore, Maryland, United States; Johns Hopkins Bloomberg School of Public Health, Baltimore, Maryland, United States; UT Southwestern Medical Center, Dallas, Texas, United States; University of Maryland School of Medicine, Baltimore, Maryland, United States; Johns Hopkins School of Medicine, Baltimore, Maryland, United States; Johns Hopkins Bloomberg School of Public Health, Baltimore, Maryland, United States; University of Minnesota School of Medicine, Minneapolis, Minnesota, United States

## Abstract

Altered circadian rest/activity rhythms (RARs), measured by accelerometers, have been linked to dementia risk, but associations with neuroimaging measures of brain health remain unclear. We studied cross-sectional associations of RARs with magnetic resonance imaging (MRI) measures of brain volumes, white-matter hyperintensity (WMH) volume, and the presence of infarcts and microbleeds in 396 cognitively unimpaired Atherosclerosis Risk in Communities Study participants (mean±SD age = 78.9±4.6; 57.1% women; 28.0% Black) who wore the Zio XT ECG monitor (containing an accelerometer) for 13.0±2.1 days at Visit 6 (2016-17) and had 3-Tesla MRI scans at Visit 6 or 7 (2016-19). RAR indices of rhythm strength (relative amplitude; RA), fragmentation (intradaily variability; IV), and day-to-day stability (interdaily stability; IS) were derived from accelerometer data. After adjustment for demographic and clinical characteristics, ApoE e4 allele carriage, and intracranial volume, each SD increase in RA was associated with 1.0% greater frontal, temporal, and parietal lobe volume, 0.9% greater total brain volume, a 13.4% smaller WMH volume, and a 28.2% lower odds of microbleeds (all p < 0.05); RA was not, however, associated with odds of an infarct (p = 0.4). In addition, higher IV was associated with lower frontal lobe volume across models (p < 0.05), but it was not associated with other outcomes in fully adjusted models. There were no significant associations of IS with MRI variables. Findings support links of weaker and more fragmented RARs with poorer brain health in cognitively unimpaired older adults. Prospective studies are needed to further evaluate the potential for a causal link.

